# ^99m^Tc-HDP bone scintigraphy and ^18^F-sodiumfluoride PET/CT in primary staging of patients with prostate cancer

**DOI:** 10.1007/s00345-017-2096-3

**Published:** 2017-10-17

**Authors:** Maurits Wondergem, Friso M. van der Zant, Remco J. J. Knol, Anne Marij G. Burgers, Siebe D. Bos, Igle J. de Jong, Jan Pruim

**Affiliations:** 1 Department of Nuclear Medicine, Noordwest Ziekenhuisgroep, Wilhelminalaan 12, 1815 JD Alkmaar, The Netherlands; 2 Department of Urology, Noordwest Ziekenhuisgroep, Wilhelminalaan 12, 1815 JD Alkmaar, The Netherlands; 3 Department of Urology, University of Groningen, University Medical Center Groningen, Groningen, The Netherlands; 4 Department of Nuclear Medicine and Molecular Imaging, University of Groningen, University Medical Center Groningen, Groningen, The Netherlands; 5 Department of Nuclear Medicine, Tygerberg Hospital, Stellenbosch University, Stellenbosch, South Africa

**Keywords:** Prostate cancer, ^18^F-sodiumfluoride PET/CT, Bone scan, Staging

## Abstract

**Introduction/Aim:**

Correct staging of patients with prostate cancer is important for treatment planning and prognosis. Although bone scintigraphy with ^99m^Tc-phosphonates (BS) is generally advised for staging by guidelines in high risk prostate cancer, this imaging technique is hampered by a high rate of inconclusive results and moderate accuracy. Potentially better imaging techniques for detection of bone metastases such as ^18^F-sodiumfluoride PET/CT (NaF PET/CT) are therefore being evaluated. In this observational cohort study we evaluate the performance and clinical impact of both BS and NaF PET/CT in primary staging of patients with prostate cancer.

**Methods:**

The first of two cohorts consisted of patients who received a BS while the second included patients who received a NaF PET/CT for primary staging of prostate cancer. For both cohorts the number of positive, negative and equivocal findings, calculated diagnostic performance of the imaging modality in terms of sensitivity and specificity, as well as the impact on clinical management were studied. The ranges of the diagnostic performance were calculated both assuming that equivocal findings were positive and assuming that they were negative for bone metastases. For the NaF PET/CT cohort the number of patients with signs of lymph node metastases on low dose CT were also recorded, including the impact of these findings on clinical management.

**Results:**

One-hundred-and-four patients underwent NaF PET/CT, whereas 122 patients underwent BS. Sensitivities of 97–100 and 84–95% and specificities of 98–100 and 72–100% were found on a patient basis for detection of bone metastases with NaF PET/CT and BS, respectively. Equivocal findings warranted further diagnostic procedures in 2% of the patients in the NaF cohort and in 16% in the BS cohort. In addition NaF PET/CT demonstrated lymph node metastases in 50% of the included patients, of which 25% showed evidence of lymph node metastases only.

**Conclusion:**

Our data indicate better diagnostic performance of NaF PET/CT compared to BS for detection of bone metastases in primary staging of prostate cancer patients. Less equivocal findings are encountered with NaF PET/CT. Moreover, NaF PET/CT has additional value over BS since lymph node metastases are encountered frequently.

## Introduction

Prostate cancer is a highly prevalent malignancy in western countries, especially in older men. Of all types of cancer in men, prostate cancer has the highest incidence, and its mortality is surpassed only by lung and colon cancer (European Cancer Observatory, http://eu-cancer.iarc.fr/). In aggressive forms of prostate cancer, haematogenous spread occurs primarily to the hematopoietic red marrow of the skeleton. The presence of such metastatic bone disease markedly influences prognosis, and early diagnosis of skeletal involvement is necessary for appropriate patient management [1].

Since the 1960s it is recognised that bone scintigraphy (BS) is capable of detecting bone metastases well before radiological change. Successful bone imaging was achieved in 1961 by Felming et al. [2] using ^85^Sr. Due to the large radiation burden and slow clearance of tracer from the blood pool this radioisotope was far from ideal. A few years later ^87m^Sr and ^18^F were introduced as alternatives for ^85^Sr [3, 4]. However, for ^87m^Sr the slow tracer extraction from soft tissues still prohibited sufficient image quality. ^18^F with its fast blood clearance and high skeletal uptake resulted in more satisfactory images. However the low availability of this isotope, which is cyclotron produced and has a relatively low half-life of 110 min, and the relatively high energy of the emitted photons of ^18^F (511 keV), which is not ideal for detection with an Anger gamma-camera, prevented its use in clinical routine. In 1971 the first radiopharmaceutical based on a phosphonate labelled with ^99m^Tc became available [5]. For that time, ^99m^Tc had ideal properties for clinical use. First, there was a good availability of this isotope, since it was used for other nuclear medicine procedures, which had become routine clinical practice and second, the relatively low photon energy (140 keV) with a half-life of 6 h was suitable for imaging with an Anger gamma-camera. Further developments resulted in introduction of ^99m^Tc-methylene diphosphonate (^99m^Tc-MDP) and ^99m^Tc-hydroxymethylene diphosphonate (^99m^TC-HDP) which are the most commonly used tracers for bone imaging with a gamma-camera [6, 7].

For decades European and US guidelines recommend bone scintigraphy with ^99m^Tc-phosphonates for bone metastasis assessment, which, if necessary can be complemented by radiographic survey [8, 9]. A closer look at the present guidelines reveals that the guidelines are mainly based on 30 years-old studies which found that BS was the most sensitive diagnostic method at that time and on arguments involving the clinical impact of BS, for which the pre-test probability for positive findings is used as a surrogate [10, 11]. The wide availability and technical improvements of Positron Emission Tomography/Computed Tomography (PET/CT) cameras after the introduction of 2-deoxy-2-(^18^F)fluoro-d-glucose (^18^F-FDG) in standard medical imaging in the late 1900s and early 2000s and the increasing insights in the diagnostic value of ^99m^Tc-MDP and ^99m^Tc-HDP bone scans, with a moderate sensitivity and poor specificity, resulted in renewed interest in ^18^F as a bone tracer in medical imaging. Although ^18^F-sodiumfluoride (NaF) PET/CT performed better than ^99m^Tc-HDP bone scan (BS) in comparative studies, data is still limited due to relatively small studied cohorts and the inclusion of mixed patient populations [12–15].

In January 2014 NaF PET/CT for detection of bone metastases was introduced in our clinical practice. Before this date BS was routinely performed. For all patients that received a NaF PET/CT on our department patient data was prospectively entered in a database for quality assessment and educational purposes. In this study we present the data of a cohort of patients that received NaF PET/CT and a cohort of patients that received BS for primary staging of prostate cancer. For both cohorts, the number of positive, negative and equivocal findings, found diagnostic performance of the imaging modality, including sensitivity and specificity, and impact on clinical management are reported. For the NaF PET/CT cohort the number of patients with signs of lymph node metastases on low dose CT are also reported, including the impact of these findings on clinical management.

## Materials and methods

Both cohorts included patients who received either BS or NaF PET/CT for initial staging of histopathologically or clinically proven prostate cancer. Clinically proven prostate cancer was defined as elevated PSA > 20 ng/ml and a clear prostate tumour by digital exam. The retrospective BS cohort included all consecutive patients from January 2011 till April 2012, while patients were included in the NaF PET/CT cohort from January 2014 till July 2016. Patients who had a second malignancy, except basal cell carcinoma of the skin, or for whom no follow-up data were available were excluded from the analysis. All patients gave written informed consent for the use of their anonymous data for scientific purposes. Besides the standard imaging protocol and standard clinical management no additional measurements or actions affecting the patient were performed. The study passed the local scientific board and approval of the local ethical committee for the present study was waived since the study does not fall within the scope of the Dutch Medical Research Involving Human Subjects Act (Sect. 1.b WMO, 26th February 1998).

Bone scintigraphy consisted of planar images of the entire skeleton, which were acquired using a dual-headed camera, 2–3 h after intravenous injection of a standard dosage of 550 MBq (range 515–589) of ^99m^Tc-HDP (General Electric Millenium™ VG3 or General Electric Millennium™ VG5 + hawkeye option). NaF PET/CT images were acquired 60 min after intravenous administration (90 s per bed position) of 2.5 MBq/kg body weight ^18^F-Sodiumfluoride (Mean 189, range 146–271) on a Siemens Biograph-16 TruePoint PET/CT (Siemens Healthcare, Knoxville, USA). Reconstruction was done by means of an iterative OSEM3D algorithm using 4 iterations and 8 subsets and a 5 mm Gaussian filter. Reconstructed images had an image matrix size of 168 × 168, a pixel spacing of 4.07 × 4.07 mm and a slice thickness of 5 mm. For attenuation correction a low-dose CT was acquired using a tube current of 25 mAs at 110 kV, collimation 16 × 1.2 mm and a pitch of 0.95. CT images were reconstructed using a slice thickness of 5.0 mm and a matrix size of 512 × 512.

All images were separately re-interpreted by two nuclear medicine physicians (MW and FZ) who were blinded to other imaging, biochemical, or histopathological findings and to the clinical follow-up. For each patient, the BS or NaF PET/CT was scored as no metastases, equivocal or positive for metastases. Cases demonstrating focal and intense tracer uptake that could not be related to benign processes were scored as malignant. Cases with irregular tracer uptake in the spine in the absence of other signs of metastases, and those with focal tracer uptake due to degenerative vertebral joints, were scored as no metastases. Rib lesions were categorized as malignant when these presented elongated uptake or when multiple randomly ordered findings of focal costal uptake was encountered, as benign when these vertically involved several ribs, and otherwise as equivocal. Low-dose CT images of the NaF PET/CT were evaluated for the presence of lymph node metastases, defined as lymph nodes with a short axis of at least 1.0 cm in the para iliac region/obturator fossa. In case of differences in image interpretation, decisive categorization was performed in consensus. According to a literature review on the role of BS in baseline staging of newly diagnosed prostate cancer by Abuzallouf et al. [16.] patients were categorised by PSA level (< 10, 10.0–19.9, 20–49.9, 50–99.9 and > 100 ng/ml) and Gleason score (≤ 7, ≥ 8 and unknown).

The used composite reference standard (RS) comprised a follow-up period of at least 18 months for the BS cohort and at least 6 months for the NaF PET/CT cohort, and included staging imaging, follow-up imaging (BS, MRI, CT, X-ray, ^18^F-fluorocholine-PET/CT, and/or ^18^F-FDG-PET/CT), biochemical follow-up, and clinical follow-up.

The diagnostic performance of each modality, including sensitivity, specificity, positive predictive value (PPV), negative predictive value (NPV) and accuracy, were calculated by comparison of the BS and NaF PET/CT results with the RS. Because both cohorts contained scans with equivocal results the ranges of the diagnostic performance were calculated both assuming that equivocal findings were positive and assuming that they were negative for bone metastases.

For all patients impact of BS and NaF PET/CT on clinical management was scored. For the retrospective BS cohort this was done by thorough survey of the electronic medical records. For the NaF PET/CT cohort this was scored during the urologic-oncologic multidisciplinary meeting. Changes in clinical management based on NaF PET/CT results were divided in changes based on findings of bone metastases and changes based on found extra-osseous metastases.

## Results

The BS cohort and NaF PET/CT cohort included 136 and 107 patients, respectively. Of these, respectively 14 and 3 patients were excluded due to a presence of an additional primary malignancy. Respectively 106 and 95 patients had histopathologically proven prostate cancer and 16 and 9 had clinically proven prostate cancer. Histopathological prove of the disease in this group was waived in case of absence of treatment options with curative intent or when patients refused from those potential curative options. Patient’s characteristics for both cohorts are presented in Table 1.

**Table 1:** Patient characteristics

	BS	NaF PET/CT
*N*	122	104
Age (years)	72.3^1^ (48–91)^2^	74.9^a^ (49–93)^b^
Gleason (*n*)		
6	6	1
7	28	16
8	30	23
9	35	49
10	9	4
Unknown	14	11
PSA (ng/ml)	28.5^c^ (1.5–3115)^b^	88.7^c^ (2.5–13500)^b^
Clinical T-score (*n*)		
1	10	3
2	26	14
3	41	49
4	7	26
Unkown	38	12

^a^Mean

^b^Range

^c^Median

**Fig. 1:**
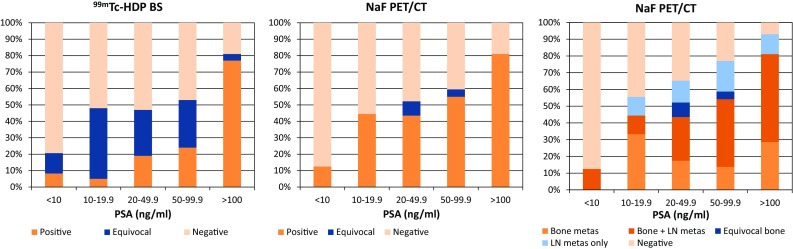
Findings on ^99m^Tc-HDP bone scan and ^18^F-NaF PET/CT per PSA category

**Fig. 2:**
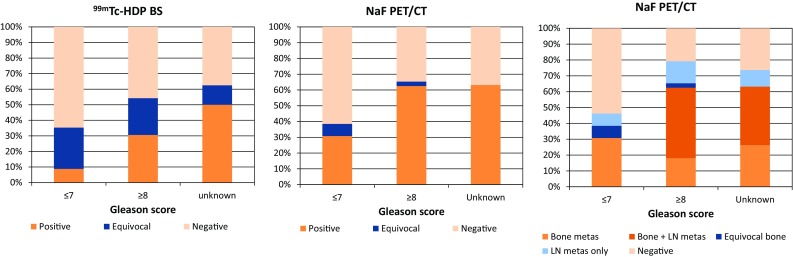
Findings on ^99m^Tc-HDP bone scan and ^18^F-NaF PET/CT per Gleason-score category

**Fig. 3:**
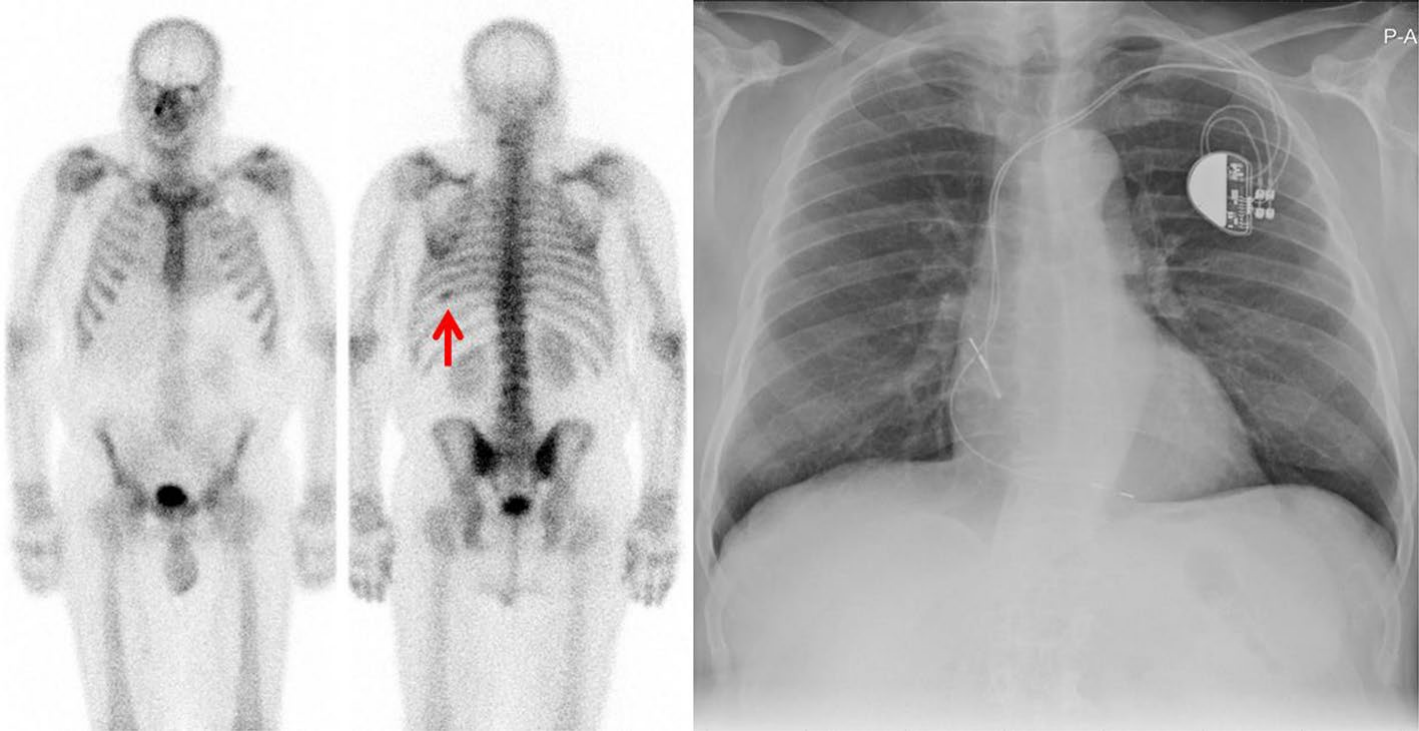
Anterior and posterior ^99m^Tc-HDP bone scan showing non-specific increased HDP uptake dorsally in the left 9th rib (red arrow). Subsequent X-thorax shows no signs of metastases. Patient received external radiation therapy with curative intent on the prostate in 2012. PSA levels decreased and PSA remained undetectable (< 0.1 ng/ml) until the latest measurement in August 2016

**Fig. 4:**
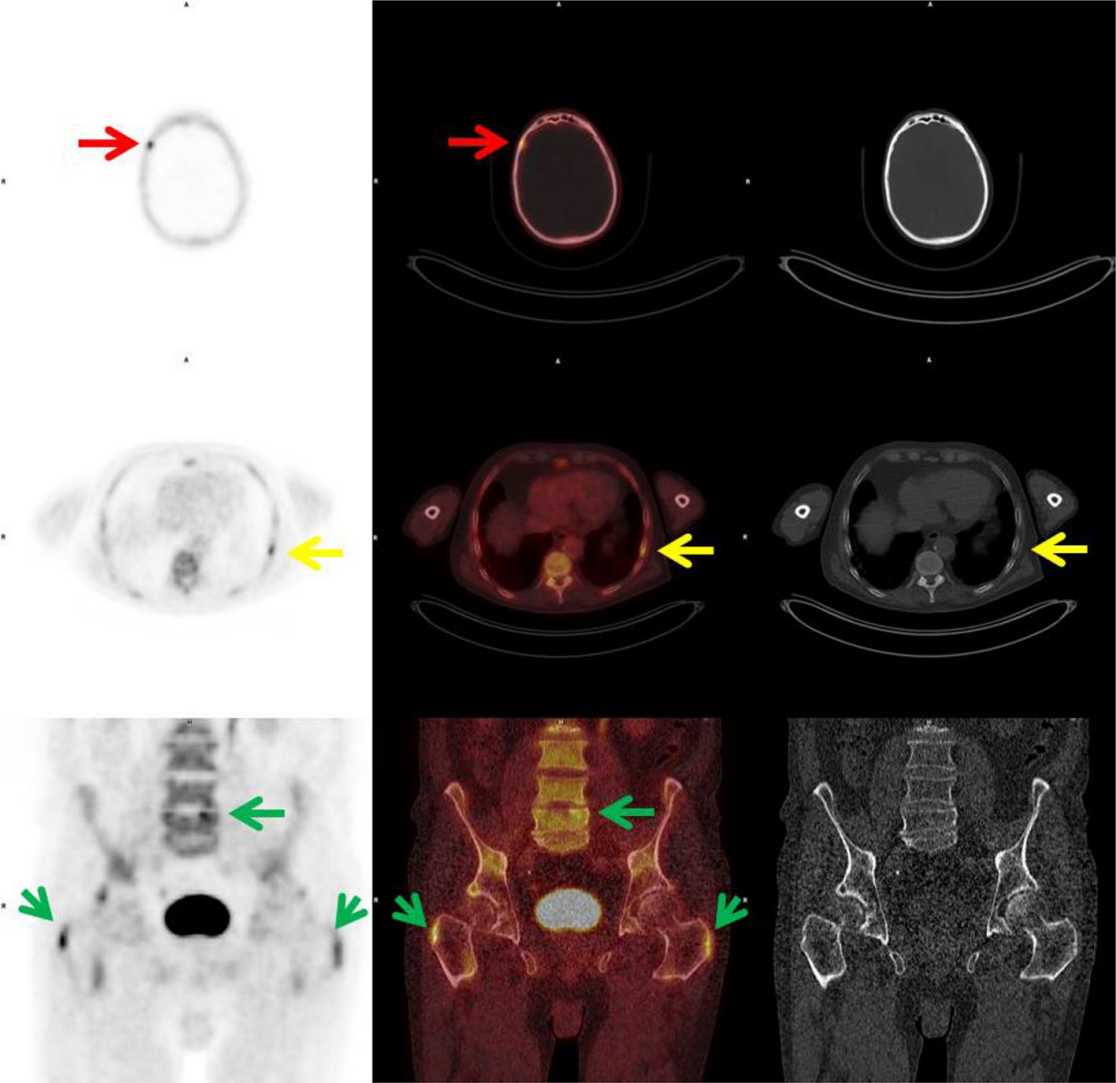
^18^F-NaF PET, PET/CT and CT images showing increased ^18^F- uptake in the right side of the os frontalis without substrate on CT, unlikely for bone metastasis (red arrows). Increased ^18^F- uptake in the right 8th rib, with faint sclerosis on CT, equivocal for bone metastasis (yellow arrows). Increased ^18^F- uptake in degenerative changes in the lumbar spine and enthesopathy in the trochanteric region at both sides (green arrows). 83-years old patient abstained from therapy with curative intent and androgen deprivation therapy was initiated

With BS and NaF PET/CT respectively, lesions characteristic for bone metastases were found in 33/122 and 61/104 patients (27 and 59%), no signs of metastases were found in 61/122 and 40/104 (50 and 39%) patients, and for 28/122 and 3/104 (23 and 3%) results were equivocal. Figure 1 shows the findings for PSA categories < 10, 10–19.9, 20–49.9, 50–99.9 and ≥ 100 ng/ml and Fig. 2 displays the findings for categories Gleason ≤ 7, ≥ 8 and unknown Gleason score. Figures 3 and 4 show examples of BS and NaF PET/CT with equivocal results. For all patients with signs of bone metastases curative options were abandoned.

For two cases in the BS cohort no RS was available: one patient died 6 weeks after BS due to cardiovascular disease, and one patient went to another hospital for a second opinion. These were excluded from further analysis. For the remaining 120 BSs and the 104 NaF PET/CTs, the imaging findings were compared with the RS and subsequently the diagnostic characteristics were calculated for both equivocal BSs and NaF PET/CTs considered positive and negative (Table 2). Found sensitivities ranged from 84.2 to 94.7% and from 96.8 to 100% for BS and NaF PET/CT and found specificities from 72.0 to 100 and 97.6 to 100%, respectively. Overall accuracy of BS ranged from 79.2 to 95.0% and for NaF PET/CT from 98.1 to 99.0%.

**Table 2:** Diagnostic characteristics of BS and NaF-PET/CT

	BS^a^ (%)	BS^b^ (%)	NaF PET/CT^a^ (%)	NaF PET/CT^b^ (%)
Sensitivity	94.7	84.2	100	96.8
Specificity	72.0	100	97.6	100
PPV	61.0	100	98.4	100
NPV	96.7	93.2	100	95.3
Accuracy	79.2	95.0	99.0	98.1
Equivocal prevalence	23.0	23.0	2.9	2.9
Disease prevalence	31.7	31.7	60.6	60.6

*PPV* positive predictive value, *NPV* negative predictive value

^a^Equivocal findings considered positive

^b^Equivocal findings considered negative

According to the reference standard 5/28 (18%) equivocal findings in the BS cohort were due to the presence of bone metastases, the other 23/28 (82%) equivocal findings were due to benign causes including degenerative disease. Equivocal findings resulted in additional imaging procedures in 19/28 patients (16% of the patients in the entire cohort) which included: MRI pelvis and total vertebral column in 10 patients, CT abdomen and pelvis in 3 patients, MRI and CT in 1 patient, X-ray of the pelvis in 2 patients and chest X-ray (including detailed costal images) in 1 patient. In the NaF PET/CT cohort 2/3 (67%) of the equivocal findings were due to bone metastases. In 2 patients (2% of the patients in the entire cohort) additional imaging was used, consisting of MRI of pelvis and vertebral column in both patients. No additional imaging was applied in the other patient who refused therapy with curative intent.

In the NaF PET/CT cohort low-dose CT images showed signs of lymph node metastases in 52/104 (50%) patients (Figs. 1c and 2c). Thirteen patients had signs of lymph node metastases only. According to the seventh TNM-classification for prostate cancer only locoregional lymphadenopathy (N1) was found in 7 of those patients. In one patient a histopathological biopsy of a lymph node near the right external iliac artery confirmed the presence of a lymph node metastasis of prostate cancer. For 5 patients, including the patient with histopathologically proven lymph node metastasis, the multidisciplinary team advised radiation therapy with curative intent combined with 3 years androgen deprivation therapy. For 2 patients palliative hormonal treatment was advised. One of those patients had a lymph node metastasis with a short axis of 7.0 cm and the other patient had severe co-morbidity. Locoregional as well as non-regional lymphadenopathy without signs of bone metastases (N1M1a) was found in 6 patients. No histopathological biopsies were taken from suspected lymph nodes in this group. The multidisciplinary team advised palliative radiation therapy for local disease control (two T4 tumours and one T3b) in combination with androgen deprivation therapy for 3 patients and only palliative androgen deprivation for the other 3 patients.

## Discussion

As a consequence of the retrospective nature of this study, differences between both cohorts in terms of patient characteristics were observed (Table 1). The median PSA-value in the NaF cohort is higher compared to the BS cohort, and also Gleason score and T-stadium of the included patients in the NaF cohort are generally higher. As a result the prevalence of bone metastases in the NaF cohort is markedly higher than in the BS cohort (61 vs 32%). To overcome this problem the cohorts were subcategorised by PSA-value (Fig. 1) and Gleason-score (Fig. 2). In most PSA categories and all Gleason score categories NaF PET/CT detects bone metastases in a higher percentage of patients. This is also reflected by the higher sensitivity of NaF PET/CT (Table 2). Thereby for BS a trade-off between sensitivity and specificity is found, while this trade-off is almost negligible for NaF PET/CT, which results in an overall high accuracy of NaF PET/CT compared to the moderate to high accuracy of BS. In all subcategories BS shows higher numbers of equivocal findings on a per patient basis compared to NaF PET/CT.

The presence or absence of metastasised disease at the time of initial diagnosis of prostate cancer has large consequences on clinical management. According to most guidelines bone metastases preclude therapies with curative intent, however this perception is shifting. There is growing evidence that an intermediate oligometastatic state has favourable survival outcomes compared to patients with widespread metastatic disease. Some patients may be curable with aggressive multimodality treatments [17]. For oligometastatic bone metastases stereotactic body radiation therapy has been shown to be safe and well tolerated with high local control rates [18–20]. In this perspective imaging techniques with higher diagnostic performances, with both high sensitivity and high specificity become even more important for proper selection and evaluation of patients suited for these aggressive therapies.

Our findings are generally in line with previous studies, which have shown a high sensitivity and specificity for NaF PET/CT and in comparative studies NaF PET/CT performed better than BS. However literature remains scarce and most studies included both patients for staging and restaging of prostate cancer and included a smaller number of patients as compared to the cohorts in present study.

In a prospective study from 42 patients with prostate cancer, Langsteger et al. found a high diagnostic performance of NaF PET/CT with a sensitivity and specificity of 91 and 83%, respectively [14]. In 24 prostate cancer patients Even-Sapir and co-workers found a patient based sensitivity and specificity of 70 and 57%, respectively, for planar bone scintigraphy compared to 100% sensitivity and specificity for NaF PET/CT [13]. In this study NaF PET/CT was also compared to ^99m^Tc-MDP SPECT, which reached a sensitivity of 92% and a specificity of 82%. Found differences in sensitivity and specificity between BS or SPECT and NaF PET/CT were significant (P < 0.05). In 49 prostate cancer patients Damle et al. found a sensitivity of 100 and 96.9% and a specificity of 70.6 and 41.2% % for detection of bone metastases with NaF PET/CT and BS, respectively. The overall accuracy was 90% for ^18^F-fluoride PET/CT and 78% for BS [12]. Poulsen et al. compared NaF PET/CT with BS in 50 patients for detection of spine metastases in 50 patients [15]. On a lesions basis (526 lesions included) they found a sensitivity and specificity of 51 and 82% for BS and 93 and 54% for NaF PET/CT, respectively. The low specificity of NaF PET/CT was attributed to the high number of false-positive lesions due to degenerative and inflammatory lesions. The low specificity found by the group of Poulsen is in contrast to the found high specificities found in other studies. As showed by our data the combination of NaF PET with CT is able to accurately differentiate benign from malignant NaF uptake, since benign uptake is commonly found in joints, between vertebral bodies and at sites of tendon insertions in contrast to bone metastases, which are normally found inside bones, at sites in the skeleton were bone marrow is present. Also findings on CT contribute to differentiation. Degenerative findings such as osteophytes and joint narrowing and calcifications in muscle tendons relate increased NaF uptake to benign processes with high accuracy.

In our cohort low-dose CT showed lymphadenopathy in the pelvis in 50% of patients. In 25% of these patients evidence of lymph node metastases was found without evidence of other metastases and in most cases this resulted in changes in clinical management. This shows that NaF PET/CT has, in addition to better detection of bone metastases, additional clinical value over BS in terms of detection of lymph node metastases. When detection of soft tissue metastases becomes more important in the primary staging of prostate cancer, given the options to treat oligo metastatic disease, PET/CT with other tracers, for example ^68^Ga- or ^18^F-PSMA, would probably be more suited than NaF PET/CT. However, evidence is needed that PSMA PET/CT is able to diagnose bone metastases with at least the same accuracy as NaF PET/CT.

In literature high specificity of a contrast enhanced CT for detection of lymph node metastases is reported, while the sensitivity is generally poor [21–24]. For detection of enlarged lymph nodes the use of a low-dose CT without intravenous contrast agents is insufficient and pathological lymph nodes may have been missed in our cohort, which is a drawback of the presented data. The recently published European Guidelines on prostate cancer instructs to perform at least cross-sectional abdominopelvic imaging (MRI or CT) and BS for intermediate-risk and high-risk prostate cancer. This would support the use of a diagnostic CT with an intravenous contrast agent [9]. Thereby NaF PET/CT can be used as a modality for both bone scan and cross-sectional abdominopelvic imaging, except for those patients for which MRI is indicated to stage the primary tumour.

A limitation of this study is the inability to compare with specific numbers how NaF PET/CT placed patients into different treatment strategies as compared to BS, which is a result of the character of the study. The comparison of two different cohorts prevents specification of the additional value that one scan has above another on the initated treatment in all patients.

Concerning costs, a general comparison of the expenses involved with BS and NaF PET/CT is hard to make. These costs are highly dependent on the facilities within a medical center or in the local area. PET scans tend to be more expensive than conventional scintigraphic procedures. However a tendency is seen in which PET scans and especially PET radiopharmaceuticals become less expensive due to several factors, including decreased required doses of radiopharmaceuticals and decreasing costs of PET radiopharmaceuticals. On the other hand, investments needed for replacement of aged existing reactors needed for production of ^99m^Tc containing radiopharmaceuticals, may result in increased expenses involved with conventional BS. As a result the use of PET/CT imaging for bone metastases may become worthwhile, especially when the benefits for the patient, including better placement of patients into treatment strategies are also taken into account.

This study is limited in the ability to compare, with specific numbers, how NaF PET/CT placed patients into different treatment strategies as compared to BS, which is a result of the character of the study. The comparison of two different cohorts prevents specification of the additional value that one scan has above another on the initiated treatment in all patients. Therefore it would be of great interest to prospectively study the impact of BS and NaF PET/CT in a single cohort, taken into account effects on patient treatment strategies and cost-effectiveness.

Concerning radiation safety of employees, there are no significant differences between the use of ^18^F-FDG and NaF. Therefore the use of NaF will fall within common practice of a well running PET department. Concerning radiation safety of patients the use of NaF PET/CT instead of BS will result in higher effective doses, which will be around 3.1 mSv for 550 MBq of ^99m^Tc-HDP and 4.5 mSv for 189 MBq of NaF [25]. Additional imaging for bone metastases is indicated in patients with high risk prostate cancer, which are most frequently treated with radiation therapy in case of localized or oligo metastasized disease or, in case of metastasized disease, with systemic treatment strategies, which at present often include docetaxel early in the course of the disease. Taken into account the side effects of those treatment strategies, the additional radiation dose of NaF PET/CT is not clinically significant.

## Conclusion

The present study shows a better diagnostic performance for NaF PET/CT as compared to BS for detection of bone metastases in primary staging of prostate cancer patients. Less equivocal findings are encountered with NaF PET/CT. Further diagnostic procedures were needed in only 2% of patients in the NaF cohort compared to 16% in the BS cohort. In addition NaF PET/CT detected lymph node metastases in half of the included patients, which resulted in alterations in clinical management in 25% of those patients.
